# Design of a Clinical Practice Guideline in Nurse-Led Ventilator-Weaning for Nursing Training

**DOI:** 10.3389/fpubh.2021.726647

**Published:** 2021-11-12

**Authors:** Sakinah Awang, Norlidah Alias, Dorothy DeWitt, Khairul Azhar Jamaludin, Mohd Nazri Abdul Rahman

**Affiliations:** ^1^Department of Curriculum and Instructional Technology, Faculty of Education, University of Malaya, Kuala Lumpur, Malaysia; ^2^Faculty of Education, Centre of Education Leadership and Policy, Universiti Kebangsaan Malaysia, Bangi, Malaysia

**Keywords:** structured advanced nursing training, mechanical ventilation, nurse-led ventilator-weaning protocol, clinical practice guideline, Fuzzy Delphi Method

## Abstract

Cardiothoracic intensive care unit (CICU) nurses have shared the role and responsibility for ventilator-weaning to expedite decision-making in patient care. However, the actions taken are based on individual's unstructured training experience as there is no clinical practice guideline (CPG) for nurses in Malaysia. Hence, this study aims to design a CPG for the process of weaning from mechanical ventilation (MV) for a structured nursing training in a CICU at the National Heart Institute (*Institut Jantung Negara*, IJN) Malaysia. The Fuzzy Delphi Method (FDM) was employed to seek consensus among a panel of 30 experts in cardiac clinical practice on the guidelines. First, five experts were interviewed and their responses were transcribed and analyzed to develop the items for a FDM questionnaire. The questionnaire, comprising of 73 items, was distributed to the panel and their responses were analyzed for consensus on the design of the CPG. The findings suggested that the requirements expected for the nurses include: (a) the ability to interpret arterial blood gases, (b) knowledge and skills on the basics of mechanical ventilation, and (c) having a minimum 1-year working experience in the ICU. On the other hand, the CPG should mainly focus on developing an ability to identify criteria of patient eligible for weaning from MV. The learning content should focus on: (a) developing the understanding and reasoning for weaning and extubating and (b) technique/algorithm for extubating and weaning. Also, the experts agreed that the log book/competency book should be used for evaluation of the program. The CPG for structured nursing training at IJN in the context of the study is important for developing the professionalism of CICU nurses in IJN and could be used for training nurses in other CICUs, so that decision for ventilator-weaning from postcardiac surgery could be expedited.

## Introduction

Mechanical ventilators (MV), which are introduced as breathing aids to save lives of critically ill patients, enable gaseous exchange for acceptable oxygen concentrations, tidal volumes, and respiratory rates to be maintained. On recovery, patients need to be weaned-off (withdrawal) the MV gradually and carefully (1–3). In fact, the weaning process should be conducted with caution and consistent to reduce the time and unexpected consequences to the patients (4–6). Also, this is to comply with the indications and prevent any complications from postcardiac surgery. This is a routine procedure among CICU nurses with interprofessional collaboration between the doctor (specialists in general and cardiac anesthesiology/intensivist) and cardiothoracic intensive care unit (CICU) nurses. This collaboration for sharing responsibilities has been documented in Malaysian practice since 1990 (7). It is also one of the several factors influencing nurse-led decision-making for the weaning process from MV.

The complexity of this process is dependent on the condition, complication, and need for MV among the patients (4). However, to maintain standards, prevent mismanagement, malpractice, unsafe treatment, any negligence, carelessness, inattentiveness, and ensure patient safety, a clinical practice guideline (CPG) is required for structured nursing training (8). As outlined by Blackwood et al. (9), the therapists or nurses should be able to: (a) establish clear criteria for patient readiness to begin weaning, (b) develop an organized guideline for therapists or nurses in reducing the patient's ventilatory support, and (c) establishing a clear set of criteria to help them decide the patient's readiness for extubation.

The collaboration for sharing responsibilities for the weaning process and the shortage of specialist doctors have led to nurses' assistance on the patients' situation to expedite medical decision-making (10). However, nurses risk medicolegal issues when performing ventilator-weaning without any structured training experience, guidelines, or evidence of documented practice. Hence, in protecting CICU nurses from medicolegal issues, a CPG for structured training to update nurses' knowledge and ability for critical thinking and clinical decision-making is required. The training requirement is part of the Nurses' Code of Conduct, which indicates that nurses can look forward to extended work rules, provided there is a structured training with a particular number of hours of credit (11).

Although in other countries, the roles and responsibilities of nurses extended to ventilator-weaning, this is not practiced in Malaysia, as there is no structured training for nurses. Currently, the CICU, IJN has allowed new nurses to perform ventilator-weaning for patients after cardiothoracic surgery under the guidance of an anesthetist, surgeon, or senior nurse. However, professional and ethical considerations are required for CPG for the process. Hence, this study aims to design a CPG for nurse-led ventilator-weaning for structured nursing training to improve knowledge and skills. This guideline would enable nurses in Malaysia to have protocols for practice and enhance their professionalism.

## Review of Literature

### Mechanical Ventilation

Mechanical ventilators can sustain patients' life in the event of inadequate spontaneous ventilation for breathing. However, the machine cannot cure the disease but only assists breathing as the patient's underlying condition needs to be corrected. Selecting the suitable mode of operation for the MV requires expertise and the focus of MV management is to ensure patients get adequate support to prevent complications.

#### The Ventilator-Weaning Process

The ventilator-weaning, or the patient's withdrawal from ventilator support and restoring spontaneous independent breathing, has a specific protocol. The protocol depends on factors such as patient's preexisting lung condition, duration of MV, and patient's general condition (physically and psychologically). The patients on MV for short periods may be weaned from ventilator support using a T-piece connected to an oxygen supply, and then, closely monitored for tolerance to the switch with assessments on vital signs and arterial blood gases (ABGs) concentration. An initial rise in respiratory rate, pulse, and blood pressure is expected but oxygen saturation and partial oxygen in the body should remain within satisfactory limits. However, patients who have been on ventilator support for longer periods may require gradual weaning from the MV.

The criteria for weaning are found in the Malaysian Ministry of Health's Program *Anaestesiologi dan Cawangan Kualiti Penjagaan Kesihatan BPP* (2006) ICU Management Protocol No. 10. The protocol is valid for general ICU patients, with intraoperative care and more than 24 h on MV. However, in IJN, cardiac cases are normally straight forward without intraoperative care, and patients were on MV for 4–6 h <24 h. Hence, this protocol is not relevant for IJN's needs.

#### Nurse-Led Ventilator-Weaning for Cardiothoracic Intensive Care

A CICU nurse's responsibility is to provide critical care to patients with potentially life-threatening heart conditions. The management of critically ill patients takes on many levels (12–14). First, improving patients' outcomes with patient-centered care, proactive management, and vigilance, coping with unpredictable events, and providing emotional support (4, 15). Second, continuous observation, so as to reduce a patient's risk of deterioration, monitor their total dependence on support equipment, and prevent their agitation or confusion that may lead to harm (16). This means assimilation, interpretation, and evaluation of information, including the patient's physical and psychological response to interventions, changes in condition, the significance of monitored physiological parameters, and the safe functioning of equipment. Lastly, communication, as the nurse is the key provider of information to patients, relatives, and other members of the interdisciplinary team. Hence, nurses in CICU are expected to be innovative, adopt new roles, multitask, and have cultural awareness. They need to be able to apply knowledge and skills to new situations to enhance the continuity, efficiency, and effectiveness of patient care (17). Thus, evidently the process of ventilation-weaning, originally the responsibility of the specialists, is done by nurses managing patients to expedite medical decision-making (10). Hence, regardless of the blurred boundary, nurses are expected to lead the ventilation-weaning perform (18).

The responsibility of ventilator-weaning allows nurses to expand their theory and practice, but nurses in Malaysia may not have sufficient evidence-based practice to absolve them from legal misconduct issues (19). In Australia, working across boundaries is common and protocols exist for the implementation of effective ventilator-weaning (16). On the other hand, some doctors believe cross-boundary work, such as ventilation-weaning in the CICU, does not require protocols as there are existing work instructions (20). Further, nurse-led ventilation-weaning saves patients' lives, as is within the scope of the objective of the CICU is to reduce the period of patients' stay and rate of infections while in the unit.

In Malaysia, the Code of Professional Conduct states that the nurses' responsibility is to protect the best interests of society. However, without structured training, nurses face the possibility of professional misconduct, as stated in the Nurses Act and Regulations, 1985 (Part V Practice and Part VI on Disciplinary Proceedings). Nurses can undertake extended work roles only when they have participated in structured training (NBM, 1998). Although there exists a protocol for anesthesiologists to employ safe procedures during the process of weaning (ICU Management Protocol No. 10, Program Anestesiologi & Cawangan Kualiti Penjagaan Kesihatan BPP, KKM, 2006), this protocol is for general cases and needs to be modified for CICU patients and for nursing training. Hence, a CPG for nurse-led ventilator-weaning is required for structured nursing training in Malaysia, in compliance with the Nurses Act and Regulations 1985 (NBM, 1998) to ensure the professionalism of critical care nurses.

### Nursing Training in CICU

Training systematically influences behaviors in changing knowledge, attitudes, and skill. In nursing, practitioners are encouraged to reflect critically on their practice and to view research as integral for quality care (21). Hence, training considers the variables influencing practice and explores different conditions for practitioners adopt to enable generating new knowledge and innovative practice (21). Nursing education and training need to build frameworks for evidence-based practice for critical care education and practice.

Training for skills development in the post basic intensive care unit, critical care unit, or trauma unit is conducted to ensure practitioners are protected from issues of malpractice due to uncertain boundaries. The Institute of Medicine has developed structured protocols in their CPG to systematically assist practitioners in decision-making for appropriate health care in specific clinical circumstances (22). The CPG is a reference against malpractice, unsafe treatment, negligence, and inattentiveness, and it is used to guide health-care providers in patient-related procedures. The CPG is a resource for the continuing professional development (CPD) for nursing education (23). In the United Kingdom, CPG is used to train health-care providers in clinical aspects for nursing education (24). However, this is uncommon for nursing education in Malaysia. Hence, for CPD in addressing workplace issues and for career development, CPG for nurses is required.

### Taba Model for Curriculum Design

The Taba model for curriculum design is suitable for designing training based on the CPG for nurse-led ventilator-weaning process for nurses to improve their knowledge and skills. This is because this model takes into account the opinions of experienced practitioners for the design of the structured training. The following seven steps of the Taba model are used in the designing of the CPG: (1) identify the needs of the learner and the expectations of society, (2) formulate the learning objectives, (3) select the learning content based on the objectives, (4) decide how the content is organized based on the learning needs, (5) select the learning experiences required, (6) determine the organization of the learning activities, and (7) identify evaluation criteria for the effectiveness of the curriculum (25). Hence, for the structured training and CPG, the objectives, content, teaching strategies, and evaluation measures would be determined (26). The experts with knowledge and experience in the health-care setting were invited to contribute to the design of the structured training for nurse-led ventilator-weaning process for nurses to improve their knowledge and skills.

### Johns' Model of Reflection

The instructional design for the structured training using the CPG for nurse-led ventilator-weaning process for nurses to improve their knowledge and skills would be based on Johns' model of reflection with the Carper's ways of knowing (27). Reflective practice is important in nursing education as nurses learn through on-the-job training. The following four fundamental patterns of knowing is suitable for nursing training: (a) empirical knowing that comes from factual knowledge, (b) aesthetic knowing arising from the awareness of the patient and the wholeness of the situation, (c) personal knowing from self-understanding and empathy toward the situation, and (d) ethical knowing arising from attitude and knowledge from ethical frameworks (27). This enables nurses to critically evaluate existing practices and determine whether there is a need for change. In addition, it promotes meaningful learning as nurses have the opportunity to critique practice based on evidence and prior knowledge.

### The Current Study

Hence, for this study, the experts' consensus for determining the requirements of a structured training for nurse-led ventilator-weaning process for nurses to improve their knowledge and skills were sought in order to answer the research questions as follows:

1. What are the requirements for nurses should have before being able to perform ventilator-weaning independently in terms of knowledge, skills, and experience; and minimum work experience?
2. How can the CPG for nurse-led ventilator-weaning process for nurses assist nurses?
3. What is required in a CPG for nurse-led ventilator-weaning process for nurses to improve their knowledge and skills in terms of:
  - a) aim
  - b) learning objectives
  - c) essential content, additional content, technique for weaning,
  - d) patient readiness for weaning, most appropriate step-on mode, and setting before weaning, and
  - e) evaluation/ assessment?

## Methodology

### Research Design

The experts' consensus can be determined using the Fuzzy Delphi Method (FDM) in order to address issues such as ambiguity and subjectivity of the experts' responses (28). The FDM uses the triangular fuzzy number and defuzzification process to represent the consensus on the information and the importance of each item (28). FDM has been used for accurately predicting future trends (29), for quantifying experts' opinions on regional and urban road safety for the improvement of road safety in the future (30), and identifying key performance indices for mobile services providers (31). Clearly, FDM is a useful method for ensuring that experts' opinions are exempt of the influence of others while also ensuring their completeness and consistency (32). Although the FDM has the potential to be used to improve nursing education, it has not been utilized much. Hence, in this study, FDM may be beneficial in determining consensus on the design of a CPG for nurse-led ventilator-weaning process for nurses to improve their knowledge and skills.

### The Criteria of Experts

The panel of experts for the FDM could comprise between 10 and 50 participants (33). In this study, a panel of 30 experts was selected for decision-making and to ensure the validity and reliability of the study; the criteria for their expertise and experience were determined. The experts had to be professionals with more than 5 years' experience in clinical practice such as critical intensive care and cardiac intensive care. Next, they need to be routinely handling and performing the ventilator-weaning process. These experts were anesthesiologists, cardiac anesthesiologists, cardiac intensivists, nurse educators, nurse managers, nurse mentors, and senior staff nurses in CICU and critical care areas. These experts mostly have Diploma in Nursing (*n* = 13, 43%) and have 11–20 years of working experience (*n* = 14, 47%). Table 1 below provides a summary of educational background and working experiences of the experts.

**Table 1:** Education background and working experiences of the experts.

	**Frequency**	**Percentage (%)**
**Education background**		
Master in doctorate or nursing	9	30
Bachelor of nursing	8	27
Diploma of nursing	13	43
Total	30	100
**Working experiences**		
5–10 years	12	40
11–20 years	14	47
21–30 years	4	13
Total	30	100

### FDM Procedure

First, consent to conduct this study has been obtained from the National Heart Institute (*Institute Jantung Negara*, IJN) Malaysia and the selected experts should be the respondents for the FDM. Then, these five experts from the different care areas were interviewed to determine the items that would be in a FDM questionnaire. The responses from the interviews were transcribed and analyzed to develop the items for the questionnaire. There were 73 items in the FDM questionnaire, which was distributed to the panel of 30 experts. Their responses were analyzed using the Fuzzy number technique to ensure reliability and validity and a five-point linguistic scale was used (see Table 2) (29). The Triangular Fuzzy Number with three mean points (m1, m2, and m3) is used to calculate the defuzzification value (DV) for the ranking of the consensus (34, 35). First, the distance between two fuzzy numbers and threshold value, *d*, was determined. When *d* ≤ 0.2, and the percentage of consensus is above 75%, this indicates consensus among panel members of the panel and the item was accepted (34, 35). Finally, the DV was calculated to rank the items.

**Table 2:** The five point linguistic scale.

**Linguistic variable**	
Strongly disagree	(0.00, 0.10, 0.20)
Disagree	(0.10, 0.20, 0.40)
Moderately agree	(0.20, 0.40, 0.60)
Agree	(0.40, 0.60, 0.80)
Strongly agree	(0.60, 0.80, 1.00)

## Findings

The findings of this study would determine the design of the structured training with the CPG for nurse-led ventilator-weaning for nurses. The analysis was employed to answer the research questions and only items that were accepted, with *d* ≤ 0.2, are shown in the tables.

### Knowledge, Skills, and Experience Required for Nurses to Be Able to Perform Ventilator-Weaning Independently

The basic knowledge required by all CICU staff before they could independently care for CICU patients was determined. This would ease the concern that junior nurses may lack the skills and knowledge for the procedure as a standard guideline with the requirements for ventilator-weaning would increase confidence (36). The experts achieved consensus with the highest ranking for the ability to interpret arterial blood gases (DV = 0.76), followed by knowledge and skill on the basics of mechanical ventilation (DV = 0.75) (see Table 3). Items that emerged during the interview such as knowledge and skill for troubleshooting for simple mechanical ventilators, knowledge on anatomy and physiology of respiratory system or cardiothoracic and cardiovascular care, and having a basic life support (BLS) certification were rejected and deemed unnecessary for CPD.

**Table 3:** Knowledge, skills, and experience required for nurses to be able to perform ventilator-weaning independently.

**Details for subitem**	**Fuzzy evaluation**	**Defuzzification value (DV)**	**Consensus**
Able to interpret arterial blood gases (ABG)	(16.8, 22.8, 28.8)	0.76	Accepted
Knowledge and skill on basic mechanical Ventilation	(16.6, 22.6, 28.6)	0.75	Accepted
Able to look after a critically ill patients in ICU	(15.5, 21.4, 27.4)	0.71	Accepted
Staff must be credentialed and privileged	(15.4, 21.4, 27.4)	0.71	Accepted

### Minimum Work Experience Required for Nurses to Be Able to Perform Ventilator-Weaning Independently

Nurses with work experience are able to undertake new roles such as ventilation-weaning to be more responsible and self-sufficient (14). The experts determined that at least 1 year's work experience in the ICU (DV = 0.57) and 6-months experience in CICU were sufficient for nurses to be able to undergo the training for ventilator-weaning (DV = 0.57) (see Table 4).

**Table 4:** Minimum work experience required for nurses to be able to perform ventilator-weaning independently.

**Details for subitem**	**Fuzzy evaluation**	**Defuzzification value (DV)**	**Consensus**
One (1) year in ICU	(16.4, 22.4, 28.4)	0.75	Accepted
Six (6) months in CICU	(11.3, 17.1, 23)	0.57	Rejected

### Function of CPG for Nurse-Led Ventilator-Weaning Process

The function of the CPG should be determined to indicate when and how it could be used. The consensus among the experts was that the CPG would be used as a guide for weaning with straightforward surgical cases (DV = 0.75), to provide a standard technique of weaning in the ICU, and ensuring the standard of care is maintained followed by staff (both with DV = 0.71) (see Table 5). However, during the interview with experts, an item emerged, which was providing a template to safely wean-off patients but this was rejected (DV = 0.67), as it was believed that this process is specific to the individual and could not be generalized with a template.

**Table 5:** Function of CPG for nurse-led ventilator-weaning process in assisting nurses.

**Details for subitem**	**Fuzzy evaluation**	**Defuzzification value (DV)**	**Consensus**
As a guide for straight forward surgical cases in process of weaning	(16.4, 22.4, 28.4)	0.75	Accepted
Provide a standard technique of weaning in the ICU	(15.4, 21.4, 27.4)	0.71	Accepted
To ensure standard of care are maintained and followed by all staff	(15.4, 21.4, 27.4)	0.71	Accepted
Provide the nurse with a correct way to wean patients	(15, 21, 27)	0.70	Accepted

### The Aim of the CPG

The aim of the CPG would determine the rationale for use. The highest consensus was for providing guidelines as a method of standardization of care for best practices in ICU (DV = 0.76), followed by being a guide for the nurse to perform the process of weaning step-by-step (DV = 0.75) (see Table 6). Items rejected were “to promote patient safety and reduce complication” (DV = 0.69), “as the correct approach for weaning off from mechanical ventilation” (DV = 0.68), “help the nurses in clinical decision making” (DV = 0.68), and “reduce weaning time” (DV = 0.59). Hence, the emphasis was on the CPG being a guide for practice.

**Table 6:** Aim of the CPG for nurse-led ventilation weaning process.

**Details for subitem**	**Fuzzy evaluation**	**Defuzzification value (DV)**	**Consensus**
Provide a guideline as a method of standardization of care for best practices in ICU	(16.8, 22.8, 28.8)	0.76	Accepted
CPG as a guide to the nurse to perform step by step in weaning	(16.4, 22.4, 28.4)	0.75	Accepted
As a reference when weaning off ventilation	(15.2, 21.2, 27.2)	0.71	Accepted

### The Learning Objectives of the CPG

The panel of experts achieved consensus on three learning objectives for the CPG with the highest ranking for nurses to be able to identify criteria of patient eligible for weaning from MV (DV = 0.76) (see Table 7). However, the item “reduce variation from common practice” was rejected (DV = 0.68) as the CPG was only a guide.

**Table 7:** Learning objectives of the CPG for a nurse-led ventilator-weaning.

**Details for subitem**	**Fuzzy evaluation**	**Defuzzification value (DV)**	**Consensus**
Must be able to identify criteria of patient eligible for weaning from mechanical ventilation (MV)	(16.8, 22.8, 28.8)	0.76	Accepted
Must be able to perform process of weaning without any risk and complication to the patient	(16.4, 22.4, 28.4)	0.75	Accepted
Must be able to identify criteria of failed weaning & extubation	(15.2, 21.2, 27.2)	0.71	Accepted

### Essential Content in the CPG

There were seven essential contents for the CPG, which emerged during the first round of interviews. All the seven contents that emerged were accepted to be included with the highest consensus for the inclusion and exclusion criteria of weaning (DV = 0.75) and the process of intubation and extubation (DV = 0.71) (see Table 8).

**Table 8:** Essential content in the CPG for a nurse-led ventilator-weaning.

**Details for subitem**	**Fuzzy evaluation**	**Defuzzification value (DV)**	**Consensus**
Inclusion and exclusion criteria of weaning & extubation	(16.4, 22.4, 28.4)	0.75	Accepted
Criteria & Technique for weaning	(16.2, 22.2, 28.2)	0.74	Accepted
Weaning algorithm	(16.2, 22.2, 28.2)	0.74	Accepted
Criteria when a nurse should escalate to Mentor/ Doctor	(16.2, 22.2, 28.2)	0.74	Accepted
Acceptable range of haemodynamic parameters during processes of weaning	(16, 22, 28)	0.73	Accepted
Intubation and extubation technique	(15.2, 21.2, 27.2)	0.71	Accepted
Understand the reason for ventilating a patient	(15.4, 21.4, 27.4)	0.71	Accepted

### Additional Content for the CPG

Other than the essential contents, the panel of experts identified six additional contents that should be included in the CPG to ensure it was relevant and effective. The highest consensus was for criteria of extubation (DV = 0.75), weaning algorithm (DV = 0.74), and hemodynamic monitoring normal parameter for weaning (DV = 0.73) (see Table 9). However, items such as having a “background section” and “basic anatomy and physiology of the respiratory system” were rejected (DV = 0.64 and 0.57, respectively). Only relevant content was prioritized for CPD, so as to achieve excellent outcomes.

**Table 9:** Additional content for the CPG for a nurse-led ventilator-weaning.

**Details for subitem**	**Fuzzy evaluation**	**Defuzzification value (DV)**	**Consensus**
Criteria of extubation	(16.4, 22.4, 28.4)	0.75	Accepted
Weaning algorithm	(16.2, 22.2, 28.2)	0.74	Accepted
Hemodynamic monitoring- normal parameter for weaning	(15.8, 21.8, 27.8)	0.73	Accepted
Inclusion and exclusion criteria	(15.6, 21.6, 27.6)	0.72	Accepted
Indication and complication	(15.4, 21.4, 27.4)	0.71	Accepted
Basic mechanical ventilation—application, mode and setting	(15, 21, 27)	0.70	Accepted

### Techniques to Be Included in the CPG

The techniques would be the skills that were needed to be included in the CPG for nurses to perform ventilator-weaning. There were six techniques identified with the highest consensus for clinical assessment (physical assessment) (DV = 0.75) followed by criteria for weaning (e.g., hemodynamic stability, low ventilator setting, resolution of the cause of intubation) (DV = 0.73) (see Table 10). However, items such as “Change to Pressure support (P/S) or Trachy Vent” and “Observe for signs and symptoms of Increase Work of Breathing such as Tachypneic, Sweating, Reduce Saturation Partial Oxygen (SPO2)” were rejected (DV = 0.69 and 0.60, respectively). Performing the right techniques would depend on the staff's experience.

**Table 10:** Techniques to be included in the CPG for a nurse-led ventilator-weaning.

**Details for subitem**	**Fuzzy evaluation**	**Defuzzification value (DV)**	**Consensus**
Clinical Assessment (Physical Assessment) before processes of weaning	(16.4, 22.4, 28.4)	0.75	Accepted
Criteria for weaning (eg. Hemodynamic stability, low ventilator setting, resolution of cause of intubation)	(15.8, 21.8, 27.8)	0.73	Accepted
Check ABG	(15.4, 21.4, 27.4)	0.71	Accepted
Decide whether patient fulfill extubation criteria	(15.4, 21.4, 27.4)	0.71	Accepted

### Strategies to Be Included in the CPG

The strategies that were recommended for the CPG would ensure the knowledge and skills were transferred to the nurses. The experts achieved consensus on conducting training and education (DV = 0.75) and rejected items such as conducting an audit, looking at other weaning protocol, and including recent research/ continuous studies for improvement (see Table 11). Some of the items rejected were related to designing and implementing the new CPG for evaluation and were not relevant.

**Table 11:** Strategies to be included in the CPG.

**Details for subitem**	**Fuzzy evaluation**	**Defuzzification value (DV)**	**Consensus**
Conduct training and education	(16.6, 22.6, 28.6)	0.75	Accepted

### Patient Information Needed to Decide Patient Readiness to Be Included in the CPG

The CPG would ensure nurses made quality and relevant decisions to prevent harm and complications to the patient. The highest consensus was for stable hemodynamic parameter (DV =0.75), as this would indicate patients' readiness for extubation, followed by determining neurological status (DV = 0.73) (see Table 12). Patient readiness was important for decision-making to avoid bringing harm to the patient.

**Table 12:** The patient information needed to decide patient readiness to be included in the CPG for a nurse-led ventilator-weaning.

**Details for subitem**	**Fuzzy evaluation**	**Defuzzification value (DV)**	**Consensus**
Stable Hemodynamic parameter	(16.4, 22.4, 28.4)	0.75	Accepted
Neurological status—Patient is full conscious/ Glasgow Coma Score (GCS)- more than 13, Rass Score (+1)—(−1)	(16, 22, 28)	0.73	Accepted
Minimal ventilation setting	(15.6, 21.6, 27.6)	0.72	Accepted
Minimal inotropic support	(15.4, 21.4, 27.4)	0.71	Accepted

### Step-On Mode and Setting Before Weaning to Be Included in the CPG

The process of discontinuing MV is divided into three phases: preweaning, weaning, and extubation (37). The assessment of physiological readiness of patients for weaning is to identify the criteria to begin the processes of weaning. The pulmonary function parameter for predicting weaning lists that parameter that can be determined at the bedside to predict weaning success. Establishing a relationship of trust and support is essential in avoiding the panic and anxiety that commonly prevents weaning attempts. There were four possible step-on modes and settings before weaning off the MV emerging for weaning, which emerged during the interviews. The expert consensus was only on one mode: Mechanical ventilation Mode SIMV with VC + PS → start reduce the FiO2 from 50 to 40% (DV = 0.75) (see Table 13). This is significant because this is a process of reducing MV support based on a weaning algorithm and would involve strategies to ensure patients received the best treatment with the fewest complications during the weaning process.

**Table 13:** Step-on mode and setting before weaning to be included in the CPG for a nurse-led ventilator-weaning.

**Details for subitem**	**Fuzzy evaluation**	**Defuzzification value (DV)**	**Consensus**
Mechanical ventilation mode SIMV with VC + PS → start reduce the FiO2 from 50 to 40% → Check ABG Result- If normal or hemodynamic normal → tail down the MV setting: Respiratory Rate until Minimal (eg., 12 until 8) and PEEP until minimal → Check ABG – If Normal → reduce to MV Mode CPAP/ PS → Initiate PSV **(**PEEP = 6–8 cmH2O, P/S = 10–14 cmH2O, reduce P/S by 1–2 cmH2O/h, Until a minimum P/S = 6 cmH2O, Vt > 6 ml/kg IBW) → Assess for extubation criteria → extubate	(16.4, 22.4, 28.4)	0.75	Accepted

### Evaluation of Training for the CPG

It is important to evaluate any training to determine the success of a program (38). However, in this case, the evaluation of the CPG needs to be done for the effectiveness of the program. The evaluation tools were determined before the implementation of the CPG but the experts' consensus were only on one evaluation tool, a log book/ competency book (DV = 0.75), which could be used as an evidence for the training in order to evaluate skills (38). Other items that emerged from the interviews were rejected by the experts as evaluation tools (see Table 14).

**Table 14:** Evaluation for the training for the CPG for a nurse-led ventilator-weaning.

**Details for subitem**	**Fuzzy evaluation**	**Defuzzification value (DV)**	**Consensus**
Log book/competency book	(16.4, 22.4, 28.4)	0.75	Accepted

## Discussions and Conclusions

The CPG was important as it provided guidelines on a method for standardization of care and best clinical practices in the CICU for nurse-led ventilation-weaning procedures. The study determined the knowledge, skill, and experience required before nurses could perform ventilation-weaning confidently according to the opinion of experts. This means that the prior skills which needed to be prioritized before training is conducted such ability to interpret the ABGs could be identified before patient readiness for extubation and techniques for ventilator-weaning including determining weaning mode and setting before extubation. Only nurses who had acquired sufficient skills in the CPG and experience (a minimum of 1 year in ICU or 6 months in the CICU) would be allowed to participate in the training.

The essential content and techniques to be applied were consolidated for the CPG for the institute in the context of the study. This process would be applicable to all nurses working in cardiac intensive care settings in Malaysia. After undergoing training, the effectiveness of training could be determined, and the experts agreed that the log book/competency book was the best evaluation tool. Nurses learnt through experience and reflected on the cases based on the ways of knowing (27).

The CPG would be a resource for the algorithm, a technique, and an essential content for nurses to perform ventilation-weaning procedures. This is extremely beneficial to the CICU nurses and especially junior nurses in managing patients' postcardiac surgery toward ventilation-weaning. Having the CPG ensures standardization and prevents malpractice as it is an evidence-based method for nurses to performing the procedure, thus reducing the hospitalization cost on patient and CICU hospital stay.

The CPG would also assist in nursing training by enabling a structured training program for nurses to recall and reflect on their previous work practices in the CICU. Hence, nurses could evaluate the existing and past practices to contemplate whether it was reliable and suitable and so advance the nursing profession. The reflective model can improve nursing education in a systematic manner and nurses can benefit from the different perspectives of reflective learning (27). Hence, this design was helpful for the practice of learning the process of weaning from MV in nursing education and will enable nurses to perform the procedure confidently and safely.

This CPG focuses on postcardiac surgery for adult patients with minimum complication and not for high-risk cases or difficult intubation, and it is only for CPD for training nurses at the CICU for the process of weaning. The CPG can be used as a guide for workshops, training, or programs, to enhance CICU nurses' knowledge and skills in this area. However, it can be a benchmark for staff competency based of CPG. Hence, in this study, a new perspective for nurse education was provided in combining Taba's model for curriculum design (25) and John's model of reflection (27) for designing a CPG. In addition, the CPG for nurse-led ventilator-weaning process for training nurses was designed with experts' consensus using the FDM for nurses' training and CPD at the National Heart Institute CICU. This CPG enables practice in the clinical area, and protects the nursing profession with strong evidence for advancing education in the nursing profession. Hence, the CPG promotes collaboration and teamwork to hasten patients' recovery process and reduce complications. Recommendations for future research can include expanding on this study by developing a teaching model for nurse-led ventilation-weaning process using interpretive structural modeling (ISM). In addition, an instructional module on nurse-led ventilation-weaning process could be developed using a design and development research framework.

The CPG clearly reflects recommendations to improve the implementation of MV process as proposed by Blackwood et al. (9), which include: (a) establishing clear criteria for patient readiness to start weaning, (b) developing an organized guideline for therapists or nurses in reducing patient's ventilatory support, and (c) establishing a clear set of criteria to help them decide the patient's readiness for extubation.

In conclusion, the CPG designed for a nurse-led ventilation-weaning process for nurses' training may change the perception of Malaysian nursing practice and professionalism in the international arena. Thus, in implementing the training for nurses before they perform the ventilator-weaning procedure will prevent any issues of medicolegality in their practice.

## Data Availability Statement

The original contributions presented in the study are included in the article/supplementary material, further inquiries can be directed to the corresponding author.

## Ethics Statement

The studies involving human participants were reviewed and approved by the Institut Jantung Malaysia (The National Heart Institute of Malaysia). The patients/participants provided their written informed consent to participate in this study.

## Author Contributions

SA, NA, and DD: conceptualization, methodology, and original draft preparation. SA, NA, DD, KJ, and MA: data curation. SA, KJ, and MA: formal analysis and writing-review and editing. All authors contributed to the article and approved the submitted version.

## Conflict of Interest

The authors declare that the research was conducted in the absence of any commercial or financial relationships that could be construed as a potential conflict of interest.

## Publisher's Note

All claims expressed in this article are solely those of the authors and do not necessarily represent those of their affiliated organizations, or those of the publisher, the editors and the reviewers. Any product that may be evaluated in this article, or claim that may be made by its manufacturer, is not guaranteed or endorsed by the publisher.
